# A Conceptual Model of the Healthy Acoustic Environment: Elements, Framework, and Definition

**DOI:** 10.3389/fpsyg.2020.554285

**Published:** 2020-10-29

**Authors:** Jing Chen, Hui Ma

**Affiliations:** School of Architecture, Tianjin University, Tianjin, China

**Keywords:** healthy acoustic environment, conceptual framework, definition, physiological demands, psychological demands, behavioral demands, criteria and standards, grounded theory

## Abstract

Noise has been proved to be a risk factor of physiological and psychological health. Therefore, creating a high-quality acoustic environment for people is particularly important. The aims of this study are to explore the basic elements, propose a conceptual framework, and identify the definition of a healthy acoustic environment. Through the method of grounded theory, 75 respondents participated in interviews. The results revealed that (1) “sound sources and acoustic environment,” “people’s demands,” “criteria and standards of a healthy acoustic environment,” “matching process,” “secondary fitting process,” “context,” and “acoustic environment quality” are the basic elements of a healthy acoustic environment; (2) “matching process” and “secondary fitting process” connect all the other categories and reflect the processes by which a healthy acoustic environment is judged; (3) based on the associations revealed in the framework, a healthy acoustic environment is defined as a supportive acoustic environment that can match people’s physiological, psychological, and behavioral demands in context, and that also fits the criteria and standards. The proposal of a conceptual model for a healthy acoustic environment can provide a new perspective on designing and establishing a high-quality acoustic environment required by people in the near future.

## Introduction

Noise is an important public health issue and is attracting a growing concern since it has negative impacts on human health and well-being (Basner et al., 2015; Dorota et al., 2018). With rapid urbanization, new noise sources (e.g., wind turbines and leisure noise) continue to appear in cities (World Health Organization [WHO], 2018), and the risk of exposure to noise is gradually increasing (Dorota et al., 2018). Long-term noise exposure data have shown that 65% of Europeans living in major urban areas were exposed to daytime noise levels greater than 55 dB and more than 20% of them were exposed to night-time noise levels greater than 50 dB (European Environment Agency [EEA], 2018), which would induce adverse effects, such as ischemic heart disease, cognitive impairment, obesity, and metabolic effects (Clark and Paunovic, 2018; Kempen et al., 2018). Therefore, how to build a healthy acoustic environment against such a background has become a pressing issue for all countries around the world.

To establish a high-quality acoustic environment, countries have chiefly focused on developing laws and regulations related to noise mitigation. An early important attempt on law enactments can be observed in the Noise Control Act of 1972, United States, which aimed to establish an acoustic environment for all Americans, free from noise that jeopardized their health and welfare. Since then, other countries and regions have also enacted laws and regulations (e.g., Ministry of environmental protection of China, 2008), among which the regulations formulated by the European Union have had the greatest impact worldwide. Such efforts were mainly reflected in the Green Paper on Future Noise Policy (European Commission, 1996) and the Environmental Noise Directive (2002). The laws and regulations served to prevent more residents from being exposed to high levels of noise to a certain degree (King and Murphy, 2016). However, the noise regulations are characterized by “passive control,” with the purpose of protecting people from adverse effects (Environmental Noise Directive, 2002). With people’s increasing requirements for health and a healthy environment (World Health Organization [WHO], 1986, 1991, 2006), whether the current acoustic environment, established under the guidance of “protecting people from being negatively affected,” can satisfy people’s demands is worthy of further discussion.

Moreover, in order to integrate the associations between environmental noise and health, numerous conceptual models (e.g., Lazarus and Folkman, 1984; Stokols, 1987; Van Kamp, 1990; Rashid and Zimring, 2008) were proposed based on psychological stress theory (Lazarus, 1966). However, these models focused on revealing the impact mechanism of environmental noise on non-auditory health. The specific health dimensions and acoustical indicators that should be considered are still not clear. Therefore, from a holistic perspective, illustrating the specific dimensions and acoustical parameters of a healthy acoustic environment are necessary in order to achieve an overall health. It is worth mentioning that these conceptual frameworks, together with other health-related researches (e.g., Baum et al., 2001; Schabracq, 2003), have laid a theoretical foundation for further study to establish a holistic and practical framework on acoustic environment and health.

Furthermore, the emergence of “soundscape” shifted the concern of acoustic research from the objective acoustic environment to subjective perceptions, and it also extended the research scope from regarding sounds as psychophysical stressors to regarding them as resources (Kang et al., 2016, 2020). Indeed, environmental sounds also have perceptible positive effects rather than negative impacts (Krzywicka and Byrka, 2017; Torresin et al., 2019). For instance, Terhardt and Stoll (1981) developed a descriptor for determining the pleasantness of noise as early as 1981. Axelsson et al. (2010) clearly identified that “pleasantness” was one of the dimensions in the model of perceived affective quality of soundscape. Botteldooren et al. (2006) proposed the embodiment of likeness to music of a soundscape. The aim of the exploring the positive dimensions of soundscapes was to build a high-quality acoustic environment to promote people’s health and well-being. To achieve this goal, the association between positive soundscapes and health-related effects was explored. The short-term health effects were reported to be related to physiological indicators, such as skin conductance level, heart rate, respiration rate, electromyogram, cardiovascular response, and saliva cortisol (Annerstedt et al., 2013; Hume and Ahtamad, 2013; Medvedev et al., 2015), while long-term psychophysical effects involved self-reported physical and mental health (Booi and van den Berg, 2012; Shepherd et al., 2013). The results showed that positive soundscapes were associated with a faster stress-recovery process and better self-reported health condition (Alvarsson et al., 2010; Aletta et al., 2018a,b; Park et al., 2020). The findings of soundscape research indicated the possibility to create a healthy acoustic environment. Although former studies have tentatively explored the relationship between positive soundscape and health, and the goal of establishing a healthy acoustic environment has been proposed (Aletta and Kang, 2019), it remains unclear what elements should be considered when we want to build a healthy acoustic environment and what people care about most when mentioning a healthy acoustic environment. The elements of most concern may be core factors in creating a healthy acoustic environment. At the same time, it is also necessary to thoroughly explore the framework and definition of a healthy acoustic environment in order to guide practical work such as policymaking and noise control for the future.

Above all, establishing a healthy acoustic environment is of great importance for ensuring the health of the population as well as promoting sustainable development of the environment. Therefore, through a grounded theory approach, this study aims to (1) explore people’s demands for a healthy acoustic environment and present the basic elements thereof, (2) propose a conceptual framework of a healthy acoustic environment, and (3) define the concept of a healthy acoustic environment.

## Materials and Methods

Grounded Theory (GT) is a sociological approach to discovering theory from data (Glaser and Strauss, 1968). With systematic procedures of data collection and analysis, the GT approach allows for substantial data, in-depth insights, and multidisciplinary participants, and it is useful for elucidating the underlying defined pattern of a certain phenomenon, which is well suited for the establishment of theoretical frameworks in initial research. Although it is a sociological method, GT has been employed, adapted, and refined in a diverse array of fields such as education, social work, and nursing (Strauss and Corbin, 1990; Charmaz, 2014), and emerging studies have proved that it is also an effective way to explore people’s understanding of the acoustic environment (Liu and Kang, 2016; Park et al., 2016; Acun and Yilmazer, 2018). Therefore, GT was used to perform this study.

### Participants

The principle of data sampling in GT is to select respondents who can provide the most informative insights on the research questions. In order to collect comprehensive and extensive opinions on a healthy acoustic environment, two types of respondents were considered: ordinary residents and professionals. Before the formal interviews, 5 ordinary Chinese residents and 3 acoustic professionals were selected as targets to conduct the interview. The pre-interview mainly involved the semistructured questions of the cognition of a healthy acoustic environment. The preliminary findings showed ordinary Chinese residents seemed to provide more personal feelings based on their daily experience, while the acoustic professionals seemed to be more capable of providing expertise-based opinions, which were all helpful for enriching categories. Therefore, in the formal interview, two types of respondents were all selected. Ordinary Chinese residents were selected and interviewed face to face on streets and in parks, offices, factories, and residential areas in the Beijing–Tianjin–Hebei region. In order to obtain more diverse viewpoints, in addition to acoustic professionals, professionals with the research or education background in medicine science, environment science, sociology, psychology, and architecture were also invited to participate in investigations. Finally, the first type of respondents comprised 44 ordinary Chinese residents (labeled as P01–P44), and the other comprised 31 worldwide professionals (labeled as P45–P75). There were 27 professionals with a research background in acoustics, among whom 3, 3, and 2 professionals had an interdisciplinary research background in sociology, psychology, and environment science, respectively. 3 professionals had a research background in medicine science, and 1 professional had a research background in healthy building. Among the 75 respondents, there were 37 males and 38 females, ranging in age from 23 to 76 years old (average age = 41).

### Interview Procedure

To start the investigation, an interview outline was created. As shown in Table 1, the interview outline mainly focused on three parts. First, the basic information of each respondent was obtained. Thereafter, the characteristics of a healthy acoustic environment and people’s expectation of a healthy acoustic environment were investigated, mainly to determine people’s understanding of a healthy acoustic environment. The final part focused on people’s opinions on the current noise policy and future policy, to understand people’s attitudes toward a healthy acoustic environment. Since ordinary residents have limited knowledge of an acoustic environment, in order to make it easier to start the investigation, two approachable questions were provided before starting the second part of the interview. It should be noted that questions were given as guides only, and additional questions would be added if respondents mentioned significant information. The respondents were encouraged to freely express their opinions relating to a healthy acoustic environment. The investigation was carried out from March to August 2019. The face-to-face interviews lasted from 8 to 30 min. Respondents voluntarily signed informed consent for their involvement in the interview and allowance of audio recording during the face-to-face interview. All respondents were informed of their right to confidentiality, anonymity, and withdrawal from the study at any time. Finally, interviews were organized into transcripts comprising a total of 79,009 words.

**TABLE 1:** Interview outline.

Category	Questions
Basic information	Name; age; gender; mainly research fields (for professionals only);
Characteristics and people’s demands	How is the acoustic environment in your daily life? (for ordinary residents only) What kind of acoustic environment do you like? (for ordinary residents only)
	As a researcher/resident, in your opinion, what features should a healthy acoustic environment possess? Please give more words to describe a healthy acoustic environment. What do you hope the healthy acoustic environment can bring for you and your family?
Attitudes	Are you satisfied with the current noise policy in your country or area? Why? Most of the noise standards and noise policies are made to avoid the adverse effects brought by noise. Do you think it is necessary to make those criteria and policies based on the standard of a “healthy acoustic environment” rather than “no harm?”

### Data Analysis

The interview transcripts were coded using multistep analysis techniques (Strauss and Corbin, 1990). Data were coded with qualitative analysis software.

Firstly, in the open coding, the verbal transcript data were broken down into labels by searching for key phrases, significant factors, and relations. Labels were then gradually conceptualized and grouped together by comparing their associations and similarities. It was worth noting that data conceptualization was not obtained immediately but developed by repeatedly comparing the labels with each other and with the newly emerging codes. Finally, categories emerged.

In axial coding, the data related to categories were constantly compared, on the one hand, to rationalize the classification of the categories and to develop their subcategories, and on the other hand, to determine how the categories were linked and crosscut. The category was compared with each other to discover any existing associations. By constant comparison, initial relationships among categories were developed, and the embryonic form of the conceptual framework was created. During the final stage of this procedure, based on the relationships identified, the *coding paradigm* (Strauss and Corbin, 1990) was used to further develop the linkages among categories.

*The coding paradigm focuses on specifying a category* (***phenomenon****), that is a central idea, an event, or a happening, in terms of the* ***causal conditions*** *that give rise to it; the* ***context*** (*its specific set of properties) in which it is embedded; the* ***intervening conditions*** *that are similar to the context; the* ***action/interactional strategies*** *by which it is handled, managed, carried out; and the* ***consequences*** *of those strategies (Smyrnova and Kang, 2010).*

Finally, in selective coding, the category that was central to the phenomenon was selected as the core category. All categories related to the core category were integrated to develop a conceptual framework and to refine the theory.

## Results

During open coding, 3133 labels were identified from the two different types of respondents. An example of the open coding process is shown in Table 2. Verbal data were broken down into labels of a1 to a39, and they were then conceptualized by comparing their associations and similarities. The labels a1, a6, a21, and a22 described the sound sources in a healthy acoustic environment, and were thus integrated into the concept aa1. This concept aa1 was then further conceptualized into “sound sources” (Aa1) together with a similar concept aa2. The labels a4, a5, a8, a14, etc. described the characteristics of a healthy acoustic environment, so they were grouped together as aa3 and then integrated into “perceived characteristics of the acoustic environment” (Aa2). Then, Aa1 and Aa2 were grouped into the category “sound sources and acoustic environment” (A1). Similarly, relation labels were also developed with the same procedures. For example, a10, a11, a18, a24, etc. described the relation between the acoustic environment and people’s demands. Therefore, the relation was defined as a matching relation (aa6, aa7, aa10) during the original conceptualization process. Then, the relation was further refined to “positive/promotive effect–matching–healthy” (Aa4), “negative effect–mismatching–unhealthy” (Aa5), and “no negative effect–matching–healthy” (Aa8) respectively in the second stage of the conceptualization process, and eventually they evolved into the category “matching process” (A3). Finally, with a similar coding process, seven categories were identified, as follows: “sound sources and acoustic environment” (A1), “people’s demands” (A2), “matching process” (A3), “context” (A4), “criteria and standards of a healthy acoustic environment” (A5), “secondary fitting process” (A6), and “acoustic environment quality” (A7).

**TABLE 2:** An example of the open coding process.

Memos		Labels	Conceptualizing data	Conceptualizing data	Categories
(*In your opinion, what features should a healthy acoustic environment possess?)* P (01): A healthy acoustic environment should cover more natural sounds than human voices and traffic noise. P (02): It should be soothing or quiet sounds, such as music with a steady rhythm, which can make me relaxed. But it is not absolutely quiet. If the environment is too quiet, it makes me feel scared, so it is unhealthy. P (32): In my opinion, a healthy sound environment is diverse. When I work, it is quiet and it is necessary to have no regular voices or conversation to ensure my work efficiency; When I take my child out for a walk in the evening, the healthy sound environment is lively and it is better with music of square dancing and chirping sounds from children running or playing, so I can relax completely; A healthy acoustic environment at night is quiet, but not completely silent, which makes me sleep well. P (66): Personally, I think a healthy acoustic environment should first meet the upper limits of the noise guidelines set by the World Health Organization. In addition, it should meet people’s subjective psychological demands, such as comfortable, pleasant, and relaxed. I think the latter is more important than the former. Finally, a healthy sound environment should not only protect people’s demands from being negatively affected, but also take on a catalytic or promotive role, such as keeping one’s pleasure and evoking positive emotions.		→ a1: Key phrases: natural sounds a2: Key phrases: less human voices a3: Key phrases: less traffic noise → a4: Key phrases: soothing a5: Key phrases: quiet a6: Key phrases: music with steady rhythm a7: Key phrases: make me relaxed a8: Key phrases: not absolutely quiet a9: Key phrases: It shouldn’t make me feel scared. a10: Relation: soothing, quiet sounds, music with steady rhythm - relaxed - healthy a11: Relation: too quiet - scared - unhealthy → a12: Key phrases: diverse a13: Significant factors: work a14: Key phrases: quiet a15: Key phrases: no regular sounds a16: Key phrases: no conversation a17: Key phrases: ensure work efficiency a18: Relation: work - quiet, no regular voices, no conversation - ensure work efficiency - healthy a19: Significant factors: take child out for a walk a20: Key phrases: lively a21: Key phrases: music of square dancing a22: Key phrases: chirping sounds from children running or playing a23: Key phrases: relax a24: Relation: go out for a walk - lively, music of square dancing, chirping sounds from children running or playing - relax - healthy a25: Significant factors: sleep at night a26: Key phrases: quiet a27: Key phrases: not completely silent a28: Key phrases: make me sleep well a29: Relation: sleep - quiet, not completely silent - sleep well - healthy → a30: Key phrases: meet upper limits of the noise guidelines a31: Key phrases: meet people’s subjective psychological demands a32: Key phrases: comfortable a33: Key phrases: pleasant a34: Key phrases: harmonious a35: Key phrases: people’s psychological demands are more important than noise guidelines. a36: Relation: acoustic environment - people’s demands (not being negatively affected) - healthy a37: Relation: acoustic environment - people’s demands (take on a catalytic or promotive role) - healthy a38: Key phrases: keep one’s pleasure a39: Key phrases: evoke positive emotions	aa1: The sound sources of a healthy acoustic environment can be natural sounds, music, and chirping sounds from children running or playing (a1, a6, a21, a22). aa2: The sound sources of a healthy acoustic environment should be less human voices, less traffic noise, and no conversation (a2, a3, a16). aa3: The characteristics of a healthy acoustic environment are soothing, steady, quiet, not absolutely quiet, not regular, lively, and harmonious (a4, a5, a8, a14, a15, a20, a26, a27, a34). aa4: A healthy acoustic environment should make people relaxed, comfortable, and pleasant (a7, a23, a32, a33, a38, a39). aa5: A healthy acoustic environment should not make people scared (a9). aa6: A healthy acoustic environment is the matching result between the acoustic environment and people’s demands. If the characteristics of the acoustic environment (e.g., soothing, quiet, and steady) can have positive or promotive effects on people’s demands (e.g., relaxation and sleep well), it is a healthy acoustic environment (a10, a24, a29, a37). aa7: A healthy acoustic environment is the matching result between the acoustic environment and people’s demands. If the acoustic environment (too quiet) have negative effects on people’s demands (security), it is an unhealthy acoustic environment (a11). aa8: With different activities (work, go out for a walk, sleep at night), people have different kinds of demands (ensure work efficiency; relax; sleep well). So they desire different characteristics (quiet, no regular voices, no conversation; lively, music of square dancing, chirping sounds from children running or playing; quiet, not completely silent; a13, a18, a19, a24, a25, a29). aa9: The healthy acoustic environment should meet people’s implicit behavioral demands: work and sleep (a17, a28). aa10: A healthy acoustic environment is the matching result between the acoustic environment and people’s demands. If the characteristics of the acoustic environment (e.g., quiet, no regular voices, and no conversation) will not cause negative effects on people’s demands (e.g., work efficiency), it is a healthy acoustic environment (a18, a36). aa11: A healthy acoustic environment should meet the limits values of the noise guidelines (a30). aa12: Both layers (limits in noise guidelines and people’s psychological demands) should be used to measure a healthy acoustic environment, and people’s psychological demands are more important than noise guidelines (a30, a31, a35).	Aa1: Sound sources (aa1, aa2) Aa2: Perceived characteristics of the acoustic environment (aa3) Aa3: Psychological demands (aa4, aa5, aa12) Aa4: Positive/promotive effect—matching—healthy (aa6) Aa5: Negative effect—mismatching—unhealthy (aa7) Aa6: Behavioral states (aa8) Aa7: Behavioral demands (aa9) Aa8: No negative effect—matching—healthy (aa10) Aa9: Standards (aa11) Aa10: Fit standards and meet people’s psychological demands (aa12)	A1: Sound sources and acoustic environment (Aa1 Aa2) a2: People’s demands (Aa3, Aa7, Aa10) a3: Matching process (Aa4, Aa5, Aa8) a4: Context (Aa6) a5: Criteria and standards of a healthy acoustic environment (Aa9) a6: Secondary fitting process (Aa10) a7: Acoustic environment quality (Aa4, Aa5, Aa8)

**FIGURE 1:**
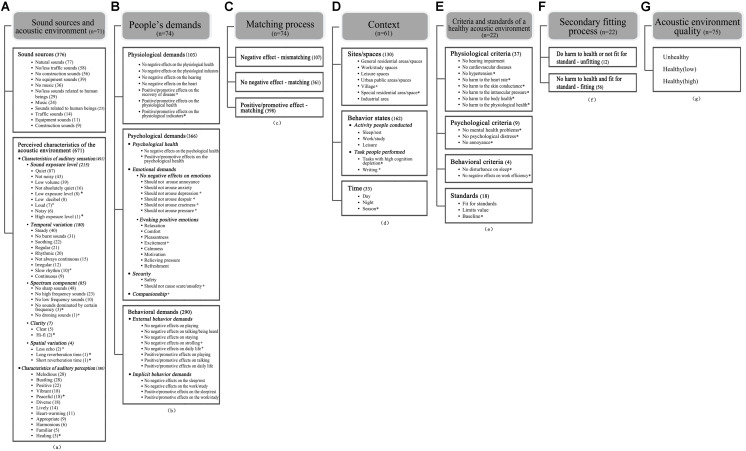
Categories, subcategories, corresponding dimensions, and key points created in the open coding process.

In axial coding, on the one hand, subcategories of each category and dimensions of each subcategory were developed. For example, “sound sources” and “perceived characteristics of the acoustic environment” were developed as two subcategories of “sound sources and acoustic environment.” Based on the conceptual data (aa3), dimensions of “perceived characteristics of the acoustic environment” were also developed; they were “characteristics of auditory sensation” and “characteristics of auditory perception.” All codes are shown in Figure 1. The categories are presented in the gray-filled boxes, while their subcategories, dimensions, and key points are shown in other boxes below, where the subcategories are presented in bold, and dimensions are presented in bold and italics, combined with their key points listed only by bullet points. Key points were directly integrated by labels of key phrases, significant factors, and relations, while dimensions were integrated by key points. It was necessary to note that some key points were further dimensionalized. For example, the key point “quiet” fell under the dimension of “sound exposure level” and “characteristics of auditory sensation,” while “sound exposure level” and “characteristics of auditory sensation” were under the subcategory of “characteristics of the acoustic environment.” On the other hand, the *coding paradigm* (Strauss and Corbin, 1990) was used to develop relationships among categories. To illustrate coding results clearly, respondents’ direct quotations, which were all listed in Table 3 except for special notes, were included. A detailed explanation of categories and the causal inference among them are presented in the following section “Elements of the Healthy Acoustic Environment.”

**TABLE 3:** Respondents’ quotations.

P01	People have different tastes. So it is better to have no music in a healthy acoustic environment.
P02	When I am sleeping, it is quiet, and there are no loud or regular sounds. If I can sleep well, it is a healthy acoustic environment.
P03	I used to live near the railway. I could not stand the rumbling noise. When the train passed, it startled me. I was annoyed and despairing. A healthy acoustic environment should not be annoying and anxiety-provoking.
P06	When I am doing high cognitive tasks, the droning sounds from the air-conditioner and chatting sounds from colleagues always distract me. These sounds should not appear in a healthy acoustic environment. It has a serious negative impact on our work efficiency.
P08:	The acoustic environment in my office is unhealthy. When I want to concentrate on my work, there is always chatting from colleagues and traffic sounds from outside. Therefore, I cannot focus my attention entirely on my work.
P11	When I am shopping in the mall, I cannot feel the sounds from the equipment at all. Even if I could hear them, the acoustic environment with equipment sounds and human voices should be lively and exciting. This is also a healthy acoustic environment, in my opinion.
P13	I really don’t want to hear traffic sounds, but I can’t make the road disappear, and I can’t change my place of work either. In my opinion, at an appropriate volume, the acoustic environment with traffic noise can also be a healthy acoustic environment.
P15	Once at the Convention Center, I talked to my client about cooperation. It was noisy. He couldn’t hear me clearly and I needed to raise my voice to make myself heard. I was anxious. In my opinion, a healthy acoustic environment should have positive effects on our behavior, and it should not affect our talking and emotions.
P16	Noise has become a disaster that threatens our health because some negative effects have been proved. Maybe our bodies have been damaged before we know it, which is terrible. I do not want such things to happen to us. A healthy acoustic environment must protect our bodies from being negatively affected.
P24	I am often at home alone, so I would like to listen to some music. Even if I don’t listen, this sound is always with me. I needed to be accompanied, and it did that. In my opinion, it is a healthy acoustic environment.
P25	In parks or other public places, if the environment is too quiet, it is frightening, and it makes people feel insecure. A healthy acoustic environment should provide a sense of security for us, especially in an empty and silent environment.
P33	A healthy acoustic environment should be different from the general acoustic environment. It should promote people’s health. I think I’m in a healthy acoustic environment now, with birds’ singing, people chatting, and laugher. I take my wife here with the hope that she can recover soon. I think a healthy acoustic environment should be able to help disease recovery.
P36	In my opinion, it is necessary to follow the current policy when establishing a healthy acoustic environment, and no disturbance in our daily life is also necessary. In particular, cars should not be allowed to whistle in the community.
P54	No effect of noise is impossible. Even if noise is not heard, people claim to be annoyed. So, some baseline for noise effects, for example, the WHO guidelines, is necessary. In order to create a healthy acoustic environment, it is necessary not only to avoid an unhealthy environment but also to preserve a pleasant environment.
P59	First of all, a healthy acoustic environment should not cause hearing impairment, or physical and mental health problems. Additionally, it should be a positive acoustic environment, and have a positive effect on people’s health, like positive soundscapes.
P62	A healthy acoustic environment depends on the site. I used to study the acoustic environment in hospitals. Quiet is necessary in a hospital because the patient needs to rest for recovery. If the acoustic environment interferes with patients’ recovery, it’s unhealthy. While a healthy acoustic environment in an urban park should be lively, pleasant, relaxing, and stress-relieving. I think the latter acoustic environment can be defined as a healthy acoustic environment in a broad sense. As x has studied, the acoustic environment in the classroom with a restorative effect on children’s attention can also be treated as a healthy acoustic environment.
P67	As a first step, healthy acoustic environments are those that “do not cause harm to health.” This could be either physiological (e.g., cardiovascular, etc.) or simply psychological distress. When adverse health effects have been addressed and excluded, the second layer comes into play, which is about a “supportive” acoustic environment, i.e., those that do not only “permit,” but basically “promote” well-being and quality of life.

**FIGURE 2:**
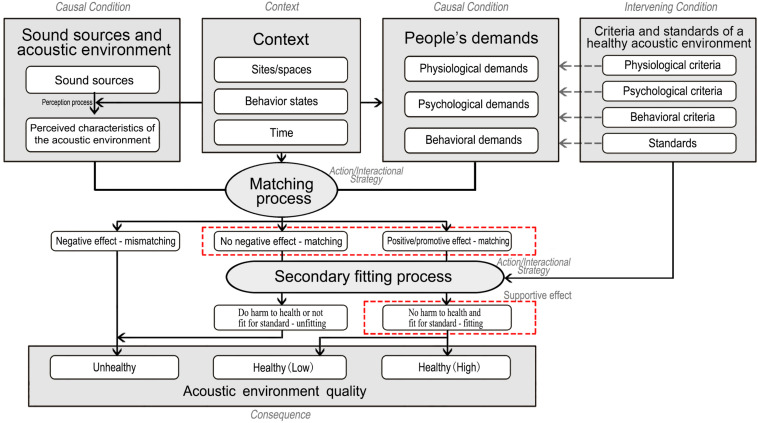
The conceptual framework of a healthy acoustic environment.

In selective coding, a conceptual framework (Figure 2) was finally created, which reflected the defined pattern of a healthy acoustic environment. The conceptual framework and the definition of a healthy acoustic environment are presented in section “Conceptual Framework and Definition of the Healthy Acoustic Environment.”

### Elements of the Healthy Acoustic Environment

#### Sound Sources and Acoustic Environment

The central idea (*phenomenon*) of this research could be labeled as the judgment of the acoustic environment quality. The category of “sound sources and acoustic environment” could be divided into two subcategories: “sound sources” and “perceived characteristics of the acoustic environment.” The sound sources were the basis of people’s perception, while gradual perception and interpretation of the acoustic environment were necessary conditions to motivate the judgment of the acoustic environment quality. Therefore, “sound sources and acoustic environment” was the first *causal condition* that gave rise to the *phenomenon*.

The “sound sources and acoustic environment” consisted of key points related to the characteristics of a healthy acoustic environment. To illustrate the characteristics of a healthy acoustic environment clearly, the frequency of labels was taken into consideration.

The “sound sources” were supported by six types of sound sources, which were natural sounds, traffic sounds, construction sounds, equipment sounds, music sounds, and sounds related to human beings. As listed in Figure 1A, the sound sources of a healthy acoustic environment can be diversified and have many manifestations. The frequencies of labels showed that people preferred natural sounds to traffic sounds, construction sounds, and equipment sounds in a healthy acoustic environment. Conflicting views were observed on music sounds and sounds related to human beings. Some respondents mentioned that music should be noted in a healthy acoustic environment while others showed a negative attitude toward music because it was difficult to find a music genre that everybody would appreciate (e.g., P01). It was also observed that sound sources in a healthy acoustic environment were closely related to people’s behavior states. For example, when people were at work with high cognition depletion, human voices in the environment could not be accepted (e.g., P06). However, when people were at leisure, human voices in the environment were considered lively and positive (e.g., P11). Interestingly, different opinions on traffic sounds, equipment sounds, and construction sounds were also found in this study, although those sounds were generally perceived as negative sound sources. As P13 mentioned, it was unrealistic to establish an acoustic environment that did not contain any traffic sounds; thus, some sound sources that people did not like could also be tolerated in a healthy acoustic environment if the sound volume was controlled at an appropriate level. Therefore, although natural sounds were preferred most, other sound categories could also be acceptable in a healthy acoustic environment, and this depended on specific context, such as people’s preferences, people’s behavior states, and some realistic conditions.

The “perceived characteristics of the acoustic environment” could be divided into two dimensions according to people’s degree of interpretation of the acoustic signal (ISO 12913-1, 2014). These were “characteristics of auditory sensation” and “characteristics of auditory perception.” “Characteristics of auditory sensation” consisted of the direct and preliminary descriptive words mentioned by people (e.g., *quiet* and *not noisy*). According to respondents’ descriptive words, five parameters of the acoustic environment were identified: sound exposure level, temporal variation, spectrum component, clarity, and spatial variation. As shown in Figure 1A, the first prominent characteristic of a healthy acoustic environment was low exposure level, because labels of *quiet*, *not noisy*, *low volume*, *low exposure level*, and *low decibel* were mentioned much more than the others labels related to sound exposure level. In addition, mild temporal variation was another characteristic of a healthy acoustic environment, which were mainly supported by labels of *steady*, *no burst sounds*, and *soothing*. Other characteristics, such as *regular*, *rhythmic*, and *not always continuous*, also seemed to be related to temporal variation of sounds. Thus, temporal variation of sounds should be considered in a healthy acoustic environment. Moreover, participants frequently mentioned that the sounds in a healthy acoustic environment should *not be sharp*. Labels of *no high frequency sounds*, *no low frequency sounds*, *no sounds dominated by certain frequency*, and *no droning sound* were also mentioned by participants. Therefore, sounds obviously dominated by certain spectrum components should be avoided in a healthy acoustic environment. Lastly, *clear*, *hi-fi*, *less echo*, *long reverberation time*, and *short reverberation time* were also mentioned by a few respondents, suggesting that clarity and spatial variation of sounds might also be parameters that should be considered in a healthy acoustic environment. “Characteristics of auditory perception” consisted of descriptive words that focused on further interpretation of the “characteristics of auditory sensation” in context, such as *melodious*, *positive*, and *bustling*. As shown, the descriptive words contained more personal positive emotions, which reflected people’s positive expectation on a healthy acoustic environment.

#### People’s Demands

By analyzing the verbal data of the interview, it was observed that when respondents referred to a healthy acoustic environment, their demands were unconsciously mentioned, such as *sleep well* (P02) and *ensure work efficiency* (P32, in Table 2, the first column). As described by respondents, the expected characteristics of a healthy acoustic environment depended on people’s demands. If their demands were met by the acoustic environment, the acoustic environment was judged as healthy. Therefore, “people’s demands” was regarded as the other *causal condition* that gave rise to the judgment of the acoustic environment quality (*phenomenon*). In this study, “people’s demands” consisted of three subcategories: physiological, psychological, and behavioral demands.

Physiological demands were composed of descriptive words related to physiological health. During the interview, because respondents did worry that the acoustic environment would negatively impact their physiological health, they expected that a healthy acoustic environment could protect them from being negatively affected (e.g., P16); thus, the key points of *no negative effects on the physiological health, no negative effects on the hearing etc.* were mentioned. In addition, some respondents also mentioned that a healthy acoustic environment should not only protect people’s physiological health from being negatively affected but also have a positive or promotive effect on people’s physiological health. For example, the key point of *positive effects on the recovery of disease* was mentioned by P33. All the key points mentioned by respondents are listed in Figure 1B.

Psychological demands consisted of demands for psychological health, emotion, companionship, and security. As listed in Figure 1B, demands for psychological health were supported by key points of *no negative effects on the psychological health* and *positive/promotive effects on the psychological health*. In addition to the general demands on psychological health, people were used to adopting a specific negative or positive emotional change to evaluate whether the acoustic environment was healthy (e.g., P03). On the one hand, some respondents (e.g., P03, P15) mentioned past experiences of negative emotions caused by the acoustic environment, so they held the opinion that a healthy acoustic environment should not arouse negative emotions (e.g., *annoyance*, *anxiety*, and *depression*). On the other hand, respondents also mentioned (e.g., P62; P66, in Table 2, the first column) that a healthy acoustic environment should evoke positive emotions (e.g., *relaxation*, *comfort*, and *pleasantness*). Moreover, some respondents mentioned demands for security (e.g., P25) and companionship (e.g., P24) when referring to a healthy acoustic environment. Demand for security was composed of descriptive phrases of *safety* and *should not cause scare/unsafety*, while demand for companionship was composed of descriptions of people’s expectations to be accompanied by sounds.

Behavioral demands were also mentioned by respondents, and they could be divided into external behavioral demands and implicit behavioral demands according to Watson’s (1913) behavioral psychology. External behaviors could be directly observed, such as *playing* and *talking*. Compared to external behaviors, implicit behaviors could only be observed with the help of equipment or experiments. In this study, the implicit behavior demands included demands on *sleep*, *rest*, *work*, and *study*. The former two demands should be a concern in situations where people need to rest, and the latter two in cases where people need to focus attention and thinking. Consistent with physiological and psychological demands, behavioral demands also had two layers: no negative effects and positive/promotive effects. All the key points mentioned by respondents are listed in Figure 1B.

#### Matching Process

According to the respondents’ description logic, the health of an acoustic environment was depended on whether the “perceived characteristics of the acoustic environment” could meet “people’s demands.” For example, as P02 described, if the perceived acoustic environment (*quiet*, *no loud or regular sounds*) could meet the demands (*sleep well*), that acoustic environment would be evaluated as healthy. In contrast, as P08 described, if the perceived acoustic environment (*colleague chatting sounds and traffic sounds*) could not meet the demand (*focus attention on work*), it would be judged as unhealthy. Therefore, the relation between “sound sources and acoustic environment” and “people’s demands” was gradually conceptualized as a matching relation (aa6, aa7, and aa10 in Table 2, the third column). Matching process reflected the process by which the judgment of the acoustic environment quality (*phenomenon*) was handled and carried out. Therefore, the matching process was considered as *action/interactional strategy* in terms of the *coding paradigm*.

The “matching process” was supported by 74 samples in this paper, and it contained three subcategories with a causal relationship. The subcategories were the cognitive outputs of “matching process,” namely “negative effect—mismatching,” “no negative effect—matching,” and “positive/promotive effect—matching.” “Negative effect—mismatching” was supported by relation labels related to the “negative effects” of the acoustic environment (e.g., a11 in Table 2, the second column), while “no negative effect—matching” and “positive/promotive effect—matching” were, respectively supported by relation labels that were related to “no negative effects” (e.g., a18 and a36 in Table 2, the second column) and “positive/promotive effects” of the acoustic environment (e.g., a10, a24, a29, and a37 in Table 2, the second column).

#### Context

In the interviews, people used to link their demands on a healthy acoustic environment and the characteristics of a healthy acoustic environment to the context. For example, as described, during *working*, *leisure*, and *sleeping* (different behavior states), the demands of P32 were to *ensure working efficiency*, *relax*, and *sleep well*, respectively, and expected characteristics of the acoustic environment correspondingly were *quiet*, *lively*, and *quiet, but not completely silent*, which showed in different “behavior states,” people had different types of demands and the characteristics that could match their demands were also different. If the characteristics of the acoustic environment could meet people’s demands in a specific behavior state, the acoustic environment was evaluated as healthy. The example suggested that “people’s demands,” “sound sources and acoustic environment,” and the “matching process” were all embedded in the “behavior states.” Therefore, “behavior states” could be regarded as the *context* to the judgment of the acoustic environment quality (*phenomenon*). Similarly, the judgment was also embedded in “site/space” (e.g., P62) and “time” (e.g., P32).

The “context” was supported by 61 samples in this study, and it contained three subcategories: “sites/spaces,” “behavior states,” and “time.” “Sites/spaces” was composed of the specific site or space that people mentioned when referring to a healthy acoustic environment. All the sites and spaces mentioned by people (e.g., *residential areas*, *office*, *urban park*, and *karaoke ba*r) were integrated into *general residential areas/spaces*, *work/study spaces*, *leisure spaces*, etc. “Behavior states” consisted of key points related to “the activity people conducted” (e.g., *sleep*, *rest*, and *work*) and “the task people performed” (e.g., *tasks with high cognition depletion* and *writing*). “Time” was supported by key points of *day*, *night*, and *season*. All the key points are shown in Figure 1D.

#### Criteria and Standards of a Healthy Acoustic Environment and Secondary Fitting Process

“Criteria and standards of a healthy acoustic environment” could be regarded as an *intervening condition* of the judgment of the acoustic environment quality (*phenomenon*) since it regulated cognitive outputs of the “matching process.” Under the supplements of “criteria and standards of a healthy acoustic environment,” the outputs of the “matching process,” namely, “no negative effect—matching” and “positive/promotive effect—matching,” were judged once more in order to exclude acoustic environments that did harm to people’s health or that were not fit for standards. Therefore, “criteria and standards for a healthy acoustic environment” can also be considered supplements to “people’s demands.”

The process of supplemental measurement was called the “secondary fitting process,” and it contained two subcategories with a causal relationship: “do harm to health or not fit for standard—unfitting” and “no harm to health and fit for standard—fitting.” “Secondary fitting process” also reflected the process by which the judgment of the acoustic environment quality (*phenomenon*) was handled and carried out, which had a similar role to the “matching process.” Thus, it was also considered as *action/interactional strategy*.

In this paper, the “criteria and standards for a healthy acoustic environment” was supported by four subcategories: “physiological criteria,” “psychological criteria,” “behavioral criteria,” and “standards.” “Physiological criteria,” “psychological criteria,” and “behavioral criteria” consisted of evidence-based descriptive phrases related to the effects of the acoustic environment on physiology, psychology, and behavior, such as *no hearing impairment* (e.g., P59), and *do not cause harm to health* (e.g., P67). “Standards” consisted of descriptive phrases related to standards, policies, or guidelines, such as *follow the policy* (e.g., P36), and *limits value of the noise guideline* (e.g., P66 in Table 2, the first column). All the key points mentioned by respondents are shown in Figure 1E.

#### Acoustic Environment Quality

According to respondents’ description, the acoustic environment quality was divided into three levels in this paper, namely “unhealthy,” “healthy (low),” and “healthy (high).” They were the final *consequences* of the judgment of the acoustic environment quality.

### Conceptual Framework and Definition of the Healthy Acoustic Environment

Based on the interpretation of each element, a conceptual framework of a healthy acoustic environment was developed to illustrate the relationships among the seven elements. As shown in Figure 2, “sound sources and acoustic environment,” “context,” “people’s demands,” “criteria and standards of a healthy acoustic environment,” and “acoustic environmental quality” are shown in gray-filled square boxes and their subcategories in rounded boxes inside, while “matching process” and “secondary fitting process” are shown in gray-filled elliptical boxes, and their subcategories in rounded boxes below. As interpreted in section “Elements of the Healthy Acoustic Environment,” the central idea (*phenomenon*) of this research could be labeled as the judgment of the acoustic environment quality, while the “matching process” and “secondary fitting process” reflected the processes by which the judgment of the acoustic environment quality was handled and carried out. Therefore, these two processes could be used to connect all the other categories. Based on the associations among these categories, the defined pattern of a healthy acoustic environment was identified.

In a specific “site/space,” “time,” and “behavior state,” people had specific “demands” on physiology, psychology, and behavior. If the “sound sources and acoustic environment” had a negative effect on “people’s demands,” the acoustic environment mismatched “people’s demands” and the output of “matching process” was “negative effect—mismatching.” Thus, the acoustic environment was directly judged as “unhealthy.” If the acoustic environment did not have a negative effect on “people’s demands” or had a positive/promotive effect on “people’s demands,” the acoustic environment matched “people’s demands” successfully. Thus, the outputs of “matching process” were “no negative effect—matching” or “positive/promotive effect—matching.” Then, the “criteria and standards of a healthy acoustic environment” came into play to measure the outputs complementally in order to exclude acoustic environments that did harm to people’s health or that were not fit for standards. Finally, if the acoustic environment has “negative effect” on people’s demands or “does harm to people’s health or not fits for standard,” it will be judged as “unhealthy.” If the acoustic environment has “no negative effect” on people’s demands and “does no harm to people’s health and fits for standard,” it will be judged as “healthy (low).” If the acoustic environment has “positive/promotive effect” on people’s demands and “does no harm to people’s health and fits for standard,” it will be judged as “healthy (high).” It should be mentioned that the acoustic environment with the characteristics of “no harm to health and fit for standards—fitting” and “no negative effect—matching” or “positive/promotive effect—matching,” as shown in Figure 2 with red dotted boxes, are consistent with the goals of a “supportive environment,” which suggests that the resources in the physical or social environment should meaningfully impact on people’s body, feelings, behaviors, and health (World Health Organization [WHO], 1991; Wagemakers et al., 2010; Jiang and Shen, 2018). Therefore, a healthy acoustic environment can be defined as a supportive acoustic environment that can match people’s physiological, psychological, and behavioral demands in context, and that also fits the criteria and standards.

## Discussion

### Regarding the Elements and Conceptual Framework of a Healthy Acoustic Environment

Associations between environmental sounds and negative health outcomes (Basner et al., 2015; Dorota et al., 2018) or positive effects (Alvarsson et al., 2010; Aletta et al., 2018a,b) have been investigated by researchers and institutions worldwide over the past decades. These works have made a great contribution to revealing the negative or positive effects of environmental sounds on people’s health. However, it remains unclear what elements should be considered and what people care about most when we want to build a healthy acoustic environment. With a grounded theory approach, this study explored the elements of a healthy acoustic environment. The proposal of these elements, together with their subcategories and dimensions, provided an opportunity for subsequent research on a healthy acoustic environment in a specific context.

Based on the associations among these elements, a conceptual framework of a healthy acoustic environment was developed. Compared with previous studies, the conceptual framework of a healthy acoustic environment is a framework with comprehensive considerations of acoustical parameters and people’s demands, and with wide applicability in context. Previously, studies either focused on examining the associations between acoustical environmental factors and a specific health outcome, such as stress (Rashid and Zimring, 2008), adverse birth outcomes (Nieuwenhuijsen et al., 2017), hearing loss and tinnitus (Sliwinska-Kowalska and Zaborowski, 2017), annoyance (Guski et al., 2017), sleep (Basner and McGuire, 2018; Meng et al., 2020b), and the cardio-metabolic system (Van Kempen et al., 2018), or focused on exploring the associations between health outcome and the acoustic environment with specific sound sources or specific characteristics, such as transport noise (Brown and van Kamp, 2017; Kempen et al., 2018; Van Kempen et al., 2018), wind turbine noise (Seltenrich, 2019), occupational noise (Themann and Masterson, 2019), low-frequency sounds (Abbasi et al., 2018), and high-frequency sounds (Fletcher et al., 2018). In addition, some integrated frameworks (e.g., Lazarus and Folkman, 1984; Stokols, 1987; Van Kamp, 1990; Rashid and Zimring, 2008) had been proposed and compared (e.g., Lercher, 1996). They mainly focused on the impact mechanism of environmental noise on health, and specific health dimensions were still not clear when we wanted to build a healthy acoustic environment. It still seems a challenge to clearly identify many-to-many relationships between specific acoustical environmental factors and specific health outcomes in practice and to construct an appropriate framework to guide such research. Despite the many challenges in identifying such complex relationships, it is extremely important for research to unravel such complexities if overall health is to be obtained (Zhang et al., 2019). The holistic conceptual framework of a healthy acoustic environment proposed in this research aims to support a movement in this direction.

### The Definition and Significance of a Healthy Acoustic Environment

In our study, a healthy acoustic environment is defined as a supportive acoustic environment that can match people’s physiological, psychological, and behavioral demands in context and that also fits criteria and standards. It can be seen that there are two key elements in assessing a healthy acoustic environment: “people’s demands” and “criteria and standards of a healthy acoustic environment.” Further revelation on these two elements will contribute to understanding the connotation of a healthy acoustic environment.

In terms of “people’s demands,” although the physiological, psychological, and behavioral demands determined in this study were not new and most of their dimensions have been considered and studied in former research (e.g., Andringa and Lanser, 2013; Darvishi et al., 2019; Fredriksson et al., 2019; Waye et al., 2019), some key points of psychological demands (e.g., demands on emotion, security, and companionship) and behavioral demands (e.g., demands on cognition) were first defined as terms related to health, suggesting that people’s demands for a healthy acoustic environment were not only confined to their physiological health but extended to a wider scope. Therefore, a healthy acoustic environment could be considered as a demand-oriented definition rather than being a narrow-health-oriented concept. The results support the definition of “health” from the perspective of the “acoustic environment” provided by WHO that “health is a state of complete physical, mental and social well-being and not merely the absence of disease or infirmity” (World Health Organization [WHO], 2006). Moreover, the results encourage and support future research related to health outcomes provided by soundscapes.

Moreover, interestingly, as shown in Figure 1B, frequency of labels showed that psychological demands were mentioned most, closely followed by behavioral demands, while physiological demands were mentioned least. It seems that in a healthy acoustic environment, people are more concerned about their psychological feelings and behavioral demands than physiological demands. Quantitative research with large samples is needed for further verification, but it is significant for policymakers and researchers to pay sufficient attention to people’s psychological and behavioral demands in a healthy acoustic environment.

This research has also revealed that a healthy acoustic environment should provide supportive effects on people’s physiology, psychology, and behavior rather than only protect people from being negatively affected. In fact, the positive effects of the acoustic environment on people’s physiological indices, psychological feelings, and behavioral responses have been observed in previous research. Clear-cut evidence suggests that interacting with nature sounds could evoke a reduced skin conductance level (Alvarsson et al., 2010), with a similar tendency observed for heart rate (Hume and Ahtamad, 2013; Medvedev et al., 2015). The restorative effects of positive soundscapes on people’s psychological experience (e.g., Herranz-Pascual et al., 2019; Meng et al., 2020a; Shu and Ma, 2020) and cognition aspects (e.g., Zhang et al., 2017; Gill et al., 2018; Shu and Ma, 2019) have been reported by many researchers, and the increased possibility of positive behavior triggered by the acoustic environment was also observed (Chen and Ma, 2019). Although part of the promotive effects make sense after stress induction, the evidence observed in studies also supports the restorative benefits and potential promotive effects of the acoustic environment on people’s physiological indices, psychological feelings, and behavioral responses. Whether the acoustic environment has a broader catalytic effect needs to be verified in further empirical research, to which sufficient attention should be paid in the future.

The “criteria of a healthy acoustic environment” consisted of four subcategories in this paper: “physiological criteria,” “psychological criteria,” “behavioral criteria,” and “standards.” It is worth noting that this study only defined the “criteria and standards of a healthy acoustic environment” within a limited scope, because, respondents held limited evidence on specific criteria and standards despite some of them being professionals. Furthermore, this study indicated that the “criteria and standards of a healthy acoustic environment” need to be systematically combed with the specific context because the judgment of a healthy acoustic environment is embedded in “sites/spaces,” “behavior states,” and “day-night.” Therefore, the saturation of “criteria and standards of a healthy acoustic environment” requires a systematic review based on the specific context. The aim of this study is to explore the overall framework of a healthy acoustic environment. Thus, detailed contents of the category in the framework need to be supplemented by follow-up research.

### Comparison of the Codes Between Ordinary Residents and Professionals

To collect comprehensive and extensive opinions on a healthy acoustic environment, two types of respondents were selected in this study: ordinary residents and professionals. The verbal data from these two types of respondents were coded together because their understandings of the healthy acoustic environment were all necessary to develop the saturated categories and an integrated framework. Based on their diverse opinions, the elements and conceptual framework of the healthy acoustic environment were proposed, and the connotation was also identified. It was also meaningful to highlight the different opinions on a healthy acoustic environment between ordinary residents and professionals to further understand the connotation of a healthy acoustic environment. Therefore, their verbal data were later coded separately. As shown in Figure 1, the codes only mentioned by professionals were marked with “^∗^,” while the codes only mentioned by ordinary residents were marked with “+” and the codes mentioned by both types of respondents were not marked.

The results showed that professionals provided more opinions based on their expertise. This is mainly reflected in three points. Firstly, more terminology was mentioned by professionals, such as *low/high exposure level*, *hi-fi*, and *long/short reverberation time* (Figure 1A). Secondly, it seemed it was easier for professionals to give a relatively complete and systematic evaluation system (e.g., P54, P59, P62, and P67) than for ordinary residents. Thirdly, professionals contributed more diverse and evidence-based key points to the category “criteria and standards of a healthy acoustic environment,” such as *no hypertension*, *no harm to heart rate*, and *no harm to skin conductance*, as observed in Figure 1E, which enriched the empirical evidence for a healthy acoustic environment. Compared with professionals, ordinary residents were more likely to provide key points from their experiences (e.g., P03, P11, and P24); thus, many key points related to their feelings were mentioned, such as *should not arouse depression*, *excitement*, and *should not cause scare/unsafety*, as observed in Figure 1B. These differences are likely to lead to different priorities in the framework of a healthy acoustic environment.

However, the aims of this study are to explore the elements, conceptual framework, and definition of a healthy acoustic environment for all people. Although some differences could be observed between the two groups of respondents, seven elements of the healthy acoustic environment were all mentioned by both groups of respondents and all the key points were essential parts to make up the framework. A complete framework covering all respondents’ opinions, whether they are professionals or ordinary residents, seems to be more meaningful for all people than two separate conceptual models. Therefore, an integrated framework of a healthy acoustic environment was proposed in this study.

### Practical Value to Acoustic Environmental Policy

Although countries have previously established noise guidelines and policies regarding different areas and different human activities (e.g., Environmental Noise Directive, 2002; Ministry of environmental protection of China, 2008), most are based on some specific health dimensions (e.g., annoyance and sleep) while the holistic perspective is lacking in policymaking. The results of this study show that systematic consideration of people’s demands is necessary for a healthy acoustic environment, which supports and promotes the rationalization of noise policy and lays the foundation for establishing the standards of a healthy acoustic environment in future.

In addition, current noise policy is established under the guidance of “no negative effect.” The results revealed that with people’s increasing requirements in relation to health and healthy environment, people hope the acoustic environment can play a promotive role on their physiological indices, psychological feelings, and behavioral demands, which may provide some hints on the parameters and limits values for future policymaking. It is worth mentioning that the aims of this study were to determine the elements, framework, and definition of a healthy acoustic environment. There is no meticulous exploration of the parameters of a healthy acoustic environment, which needs to be studied in a specific context in future. Parameters and their limits values can provide more practical value for acoustic environmental policy.

### Limitations

Many researchers focused on sound-related health outcomes, but there was not a consistent understanding on the connotation and framework of a healthy acoustic environment yet. This study proposed the definition and the framework with a grounded theory approach. However, there were some limitations.

Compared to other studies with grounded theory approach (Liu and Kang, 2016; Zhu et al., 2020), the duration of the face-to-face interview was short. The reason might be that the healthy acoustic environment is a new concept and it is a challenge even for professionals to respond to this topic, so ordinary residents have even fewer knowledge or opinion on this topic. To minimize this limitation, some encouraging or substitutive questions were also prepared and provided in the formal interview, in order to make it easier for ordinary residents to respond and to achieve an interview as deep as possible.

In addition, seven categories were saturated because there were not any new subcategories emerging after the 33th respondent. In order to make the dimensions and key points more saturated, additional respondents were interviewed. Around 70 samples, all the codes tended to be stable. It was worth mentioning that the saturation of the dimensions and key points in this study seemed not able to be achieved by interview approach because even experts in the acoustic environment and health could not provide comprehensive codes to the seven elements of a healthy acoustic environment without a systematic review. Therefore, the detailed contents of the subcategories require targeted research in a specific context under the guidance of the holistic framework. This needs further study through combining the qualitative research and systematic literature review of empirical researches.

Lastly, data collection and analysis process were all handled by the researchers, which made them part of the process and it may influence the integration of the codes. It is a limitation of GT and similar qualitative methods (Glaser and Strauss, 1968). In order to overcome the limitation, two researchers conducted the coding process separately, and the coding results were checked with a group of people with the background of acoustics. Moreover, this study followed the standardized procedure and analysis of GT and the researchers displayed all the key points, dimensions, subcategories, categories, and all the coding processes as detailed as possible.

## Conclusion

This paper presented a pilot study on a healthy acoustic environment. Semistructured interviews were conducted to explore the basic elements, a conceptual framework, and definition of a healthy acoustic environment. Overall, the main conclusions are as follows:

1. The elements of a healthy acoustic environment are “sound sources and acoustic environment,” “people’s demands,” “matching process,” “context,” “criteria and standards of a healthy acoustic environment,” “secondary fitting process,” and “acoustic environment quality.”
2. A conceptual framework was established based on the associations among these categories. The central idea (*phenomenon*) of this research can be labeled as the judgment of the acoustic environment quality. “Sound sources and acoustic environment” and “people’s demands” are the *causal conditions* that give rise to this *phenomenon*. “Context” is the *context* in which the *phenomenon* is embedded. “Matching process” and “secondary fitting process” are the *action/interactional strategies* whereby the *phenomenon* is handled and carried out. “Criteria for a healthy acoustic environment” can be regarded as *intervening condition* of the *phenomenon*. “Acoustic environment quality” (i.e., “unhealthy,” “healthy (low),” and “healthy (high)”) is the *consequence* of the *phenomenon.*
3. Based on the associations revealed in the framework, a healthy acoustic environment is defined as a supportive acoustic environment that can match people’s physiological, psychological, and behavioral demands in context, and that also fits the criteria and standards.

## Data Availability Statement

The interview data generated for this study are available on request to the corresponding author.

## Ethics Statement

The studies involving human participants were reviewed and approved by Academic Committee of School of Architecture, Tianjin University. The patients/participants provided their written informed consent to participate in this study.

## Author Contributions

HM and JC: research idea and study design, data collection, data analysis, and manuscript writing. Both authors contributed to the article and approved the submitted version.

## Conflict of Interest

The authors declare that the research was conducted in the absence of any commercial or financial relationships that could be construed as a potential conflict of interest.
